# The National Eye Institute 25-Item Visual Function Questionnaire (NEI VFQ-25) – reference data from the German population-based Gutenberg Health Study (GHS)

**DOI:** 10.1186/s12955-017-0732-7

**Published:** 2017-08-08

**Authors:** Stefan Nickels, Alexander K. Schuster, Susanne Singer, Philipp S. Wild, Dagmar Laubert-Reh, Andreas Schulz, Robert P. Finger, Matthias Michal, Manfred E. Beutel, Thomas Münzel, Karl J. Lackner, Norbert Pfeiffer

**Affiliations:** 1 Department of Ophthalmology, University Medical Center Mainz, Langenbeckstr. 1, 55131 Mainz, Germany; 2 Institute for Medical Biostatistics, Epidemiology and Informatics, University Medical Center Mainz, Mainz, Germany; 3 Preventive Cardiology and Preventive Medicine, Center for Cardiology, Cardiology I, University Medical Center Mainz, Mainz, Germany; 4 Center for Thrombosis and Hemostasis (CTH), University Medical Center Mainz, Mainz, Germany; 5 German Center for Cardiovascular Research (DZHK), partner site Rhine-Main, Mainz, Germany; 6 Department of Ophthalmology, University of Bonn, Bonn, Germany; 7 Department of Psychosomatic Medicine and Psychotherapy, University Medical Center Mainz, Mainz, Germany; 8 Center for Cardiology, Cardiology I, University Medical Center Mainz, Mainz, Germany; 9 Institute for Clinical Chemistry and Laboratory Medicine, University Medical Center Mainz, Mainz, Germany

## Abstract

**Background:**

To estimate the burden of diseases, it is important to consider patient-reported outcomes including Quality of Life (QoL). The aim of this study is to provide population-based reference data for the National Eye Institute 25-Item Visual Function Questionnaire (NEI VFQ-25), stratified by sex and age.

**Methods:**

The Gutenberg Health Study (GHS) is a population-based, prospective, observational cohort study in Germany, including 15,010 participants aged between 35 and 74. The baseline examination was conducted between 2007 and 2012. To overcome known shortcomings of the NEI VFQ-25, we calculated the previously proposed visual functioning scale and the socio-emotional scale based on Rasch-transformed person-level data. We present mean values, standard deviations and percentiles for age decades stratified by sex. We used a linear regression model to assess the influence of age, sex, socioeconomic status, distance-corrected visual acuity (better-seeing eye) and the absolute difference in distance-corrected visual acuity of both eyes on vision-related QoL.

**Results:**

NEI VFQ-25 data are available from 12,231 participants (82%). Both the long-form visual functioning scale (LFVFS) and the long-form socio-emotional scale (LFSES) showed a clear age dependency, with an average LFVFS score of 92.8 for men and 90.5 for women in the youngest age group and 85.7 and 83.4 in the oldest age group, and a LFSES score of 98.3 for men and 98.1 in women in the youngest and 94.7 and 94.5 in the oldest decade. The largest difference was observed between the youngest age group (35–44 years) and the 45–54 years group. Men tended to have slightly higher scores than women. In the multivariable linear regression analysis, age (per 5 years −0.42), female sex (−1.57), worse distance-corrected visual acuity of the better eye (per 0.1 increase in logMAR −2.92) and the difference between both eyes (per 0.1 increase in logMAR −0.87) were associated with a reduced LFVFS score (all *p* < 0.001). For the LFSES score, we showed that the influence of sex was minor, and that age (per 5 years −0.22), visual acuity of the better eye (−1.65), and the difference between both eyes (−0.56) were associated with a lower score (all *p* < 0.001).

**Conclusions:**

We report age- and sex-specific reference data from a large population-based study of mainly Caucasian ethnicity of two unidimensional scores based on Rasch-transformed NEI VFQ-25 data. Vision-related QoL is lower in older and in female individuals. Our results support the association of vision-related QoL not only with the distance-corrected visual acuity of the better eye but also with the difference in visual acuity between each eye. Our findings could be used as a reference for comparison in future studies addressing the influence of eye diseases on vision-related QoL.

**Electronic supplementary material:**

The online version of this article (doi:10.1186/s12955-017-0732-7) contains supplementary material, which is available to authorized users.

## Background

The aim of this study was to provide vision-related quality of life (VRQoL) reference data from a large population-based sample for unidimensional scores based on Rasch-transformed NEI VFQ-25 data, and to assess the associations of age, sex, socio-economic status, and distance-corrected visual acuity with VRQoL. Subjective perception of diseases and their impact on daily life activities are important measurements to estimate the burden of diseases. Patient-reported outcomes are also gaining importance in the evaluation of therapeutic interventions. For example, the European Glaucoma Society Terminology and Guidelines state that the goal of glaucoma treatment is “to maintain the patient’s visual function and related quality of life” [1]. There are many methods to measure health-related quality of life (QoL), for example, the “Short-Form 36” (SF-36) to assess health-related QoL in general [2]. General tools might miss aspects that are important for the assessment of health-related QoL in specific diseases. Therefore, tools for the assessment of QoL related to diseases have been developed. One of the most commonly used questionnaires to assess vision-related QoL (VRQoL) is the National Eye Institute Visual Function Questionnaire (NEI VFQ) [3, 4]. Subsequently, a version consisting of 25 questions (NEI VFQ-25) was developed to facilitate the application of the questionnaire in time-constrained settings [5]. The NEI VFQ-25 is commonly used and has been translated into several languages, including Italian, French, German, Spanish, Turkish, Chinese, Japanese, Greek, Portuguese, Arabic, and Serbian [6–15]. Although being one of the most frequently used vision-related QoL questionnaires, there is compelling evidence that unidimensionality and interval-level measurement, two of the most important requirements of such an instrument, are not met [16]. These shortcomings could be overcome by using different analytical techniques than simply summing up the scores, as initially described by the inventors of the questionnaire [17].

## Methods

### Study population

The Gutenberg Health Study (GHS) is a population-based, prospective, single-center cohort study at the medical center of the Johannes Gutenberg University Mainz in Germany [18]. The population sample was randomly drawn via local residents’ registration offices and equally stratified by sex and residence (urban/rural) for each decade of age. Exclusion criteria for participation in the GHS were insufficient knowledge of German and physical or mental inability to participate in the examinations in the study center. The response (recruitment efficacy proportion, i.e. the number of persons with participation in or appointment for the baseline examination divided by the number of persons with participation in or appointment for the baseline examination plus those with refusal and those who were not contactable) was 52.6% [19]. The baseline examination with a total of 15,010 participants aged 35 to 74 years took place from 2007 to 2012 and consisted of an ophthalmological examination, several general and cardiovascular examinations, as well as interviews and questionnaires. The ophthalmic part has been described in detail elsewhere [20]. In brief, we conducted measurements of autorefraction and distance-corrected visual acuity, intraocular pressure, visual field testing, pachy- and keratometry, and posterior segment photography. To assess VRQoL, we used the NEI VFQ-25. We included all participants who completed this questionnaire in this analysis.

### VRQoL data acquisition and analysis

Vision-related QoL was assessed using the German version of the NEI VFQ-25 [5, 7]. The German NEI VFQ-25 has been assessed for its psychometric properties and was used by various studies [7, 21–25]. The questionnaire was self-administered as a print-out at the study site. At the beginning of the GHS, the participants were allowed to take the questionnaire home in case they did not have the time to complete it at the study site and were asked to return it by mail. Subsequently this was changed; participants were requested to complete the questionnaire at the study site. Double data entry was applied to control for potential entry errors. The NEI VFQ-25 consists of 25 questions. The developers initially proposed to calculate 12 subscores and one composite VRQoL score, ranging from 0 = worst to 100 = best (http://www.rand.org/health/surveys_tools/vfq.html, last accessed 2017–03-10). Subsequent studies showed that this approach would result in test scores violating unidimensionality and interval-level measurement, both important properties of an instrument measuring QoL [12, 15, 16, 26, 27]. We therefore chose a Rasch-based approach, as previously used by various studies [17, 28, 29]. Rasch analysis allows to transform the raw questionnaire data into an interval-level scale. We used the transformation steps suggested by Pesudovs et al. [17]. The polarity of several questions was changed to ensure that a higher score means a lower performance. The response option “Stopped doing this for other reasons or not interested in doing this” was set to missing. The filter question 15 (“Are you currently driving, at least once in a while?”) and the related questions 15a (“IF NO: Have you never driven a car or have you given up driving?”) and 15b (“IF YOU GAVE UP DRIVING: Was that mainly because of your eyesight, mainly for some other reason, or because of both your eyesight and other reasons?”) were excluded. The tables Pesudovs et al. provided were used to map the raw scores to Rasch-transformed scores for each question on person-level. Instead of the initially proposed subscales and total sum score of the NEI VFQ-25, we calculated the visual functioning scale (long-form (LFVFS) and short-form (SFVFS)) and the socioemotional scale (long-form (LFSES) and short-form (SFSES)), based on a principal components analysis approach proposed by Pesudovs et al. [17]. This two-scale approach is supported by a factor analysis of the German NEI VFQ-39 [27]. For the LFVFS, the Rasch-transformed scores of questions 2, 5, 6, 7, 8, 9, 10, and 14 were summed up and transformed so that 0 corresponds to the sum that would be achieved if a participant would have answered all items with the worst performance, and that 100 corresponds to the sum of all items answered with the least reduction in performance. The scores of SFVFC (questions 2, 5, 6, 7, 8, 9), LFSES (questions 11, 13, 17, 18, 20, 21, 22, 23, 24, 25), and SFSES (questions 13, 17, 18, 20, 22, 23, 25) have been generated in a similar way.

### Ocular characteristics

As previously described, refraction and distance-corrected visual acuity were measured in both eyes using a Humphrey Automated Refractor/Keratometer (HARK) 599 (Carl Zeiss AG, Jena, Germany), starting with the right eye [20]. Distance-corrected visual acuity was measured using the built-in Snellen charts, ranging from 20/400 to 40/20 (decimal 0.05 to 2.0). Below that visual acuity, we used a visual acuity chart at a distance of 1 m up to 20/800, and then counting fingers, hand movements, and test of light perception. The spherical equivalent was calculated as the spherical correction value plus half the cylindrical power. Intraocular pressure was measured with an air-puff noncontact tonometer (Nidek NT-2000; Nidek, Co., Gamagori, Japan). Starting with the right eye, the mean of three measurements within a 3-mmHg range was obtained for each eye. History of eye diseases was assessed in a short interview preceding the eye examination.

### Risk factors and comorbidities

Diabetes mellitus was defined by fulfilling one of the following criteria: diabetes mellitus diagnosed by a physician, known therapy (oral medication or insulin), or HbA1c > =6.5%. Dyslipidemia was defined by a low-density lipoprotein (LDL) to high-density lipoprotein ratio (LDL/HDL) of >3.5, triglyceride levels after overnight fasting >150 mg/dL, lipid-lowering medication, or diagnosis by a physician. Hypertension was defined in the case of the use of antihypertensive medication, systolic blood pressure > 140 mmHg or diastolic blood pressure > 90 mmHg. Smoking was dichotomized into current smokers and non-smokers (including past smokers). Obesity was defined as a BMI > = 30 m^2^/kg. Cardiovascular disease was defined as history of ischemic heart disease, myocardial infarction, stroke, or peripheral arterial disease.

### Sociodemographic characteristics

The socioeconomic status (SES) was based on income, education and occupation and was defined according to the SES-index as used within the German Health Update 2009 (GEDA), with a range from 3 to 21 (three indicates the lowest SES and 21 the highest SES) [30]. Total years of education were summarized from school education, vocational training, and university education.

### Statistical analysis

The data management team performed quality controls for all data and checked for completeness and correctness by predefined algorithms and quality plausibility controls. Analyses were performed using SPSS version 23 (Statistical Package for the Social Sciences, Chicago, Illinois, USA) and SAS software 9.4 (SAS Institute Inc., Cary, NC, USA). For descriptive analyses, we calculated the mean of the spherical equivalent and the intra-ocular pressure of both eyes for every participant. The distribution of NEI VFQ-25 scores was presented by calculating the mean, standard deviation, range and percentiles in steps of 5%, stratified by sex and decade of age. To assess a potential non-responder-bias, we compared the characteristics of participants who completed the questionnaire to participants with missing questionnaire information. We performed linear regression models to determine the association of the NEI VFQ-25 long-form visual functioning scale (LFVFS) and the long-form socio-emotional scale (LFSES) with age, sex, SES, and distance-corrected visual acuity. We additionally present distributions and associations of the short-form scales (SFVFS, SFSES) to allow for comparison.

## Results

Of the entire GHS population, 13,217 participants filled the NEI VFQ-25, and 12,231 (81.5%) participants completed the questionnaire without missing items necessary to calculate the proposed scores. Among participants with missing VRQoL information, comorbidities and risk factors such as diabetes mellitus (13.1% vs. 8.5%), hypertension (52.6% vs. 49.1%), dyslipidemia (48.7% vs. 43.3%), smoking (24.4% vs. 18.4%), obesity (29.7% vs. 24.2%) and cardiovascular disease (13.0% vs. 8.8%) were more frequent (Table 1). The frequencies of self-reported eye diseases and intraocular pressures were similar, mean spherical equivalents (−0.3 D vs. −0.45 D) and visual acuities (0.06 logMAR vs. 0.03 logMAR) slightly higher (Table 1).

**Table 1:** Characteristics of the baseline sample of the German population-based Gutenberg Health Study (GHS), 2007–2012

		NEI VFQ-25 scales (LFVFS, SFVFS, LFSES, SFSES) available (81.5%)			NEI VFQ-25 missing, or at least one scale (LFVFS, SFVFS, LFSES, SFSES) missing (18.5%)		
		All	Men	Women	All	Men	Women
N	% (n)	12,231	50,2% (6139)	49,8% (6092)	2779	52.0% (1445)	48.0% (1334)
Age	mean (SD)	54.8 (11.1)	55.2 (11.1)	54.4 (11.1)	56.0 (11.1)	55.7 (11.1)	56.4 (11.1)
Risk factors and comorbidities:							
Diabetes mellitus	% (n)	8.5% (1034)	10.9% (666)	6.1% (368)	13.1% (361)	13.7% (197)	12.4% (164)
Hypertension	% (n)	49.1% (6008)	54.6% (3349)	43.7% (2659)	52.6% (1458)	55.0% (793)	50.0% (665)
Dyslipidemia	% (n)	43.3% (5287)	54.2% (3326)	32.3% (1961)	48.7% (1347)	56.4% (811)	40.3% (536)
Current smoking	% (n)	18.4% (2248)	19.4% (1191)	17.4% (1057)	24.0% (663)	26.9% (385)	21.0% (278)
Obesity	% (n)	24.2% (2961)	25.6% (1573)	22.8% (1388)	29.7% (822)	29.0% (418)	30.3% (404)
Cardiovascular disease	% (n)	8.8% (1072)	11.5% (707)	6.0% (365)	13.0% (360)	15.8% (228)	9.9% (7132)
Socioeconomic status	mean (SD)	13.1 (4.4)	13.9 (4.5)	12,4 (4.1)	11.8 (4.7)	12.4 (4.8)	11.1 (4.4)
Total years of education	mean (SD)	13.9 (2.9)	14.3 (2.9)	13.5 (2.8)	13.3 (3.0)	13.6 (3.0)	12.8 (2.9)
Urban residency	% (n)	46.0% (5629)	45.2% (2775)	46.8% (2854)	48.7% (1353)	49.1% (710)	48.2% (643)
Ocular parameters:							
Wearing glasses	% (n)	87.4% (10,688)	86.3% (5296)	88.5% (5392)	87.5% (12,431)	85.6% (1237)	89.5% (1194)
Mean spherical equivalent (diopter)	mean (SD)	-0.45 (2.44)	−0.47 (2.38)	−0.43 (2.51)	−0.30 (2.41)	−0.31 (2.33)	−0.29 (2.54)
Mean intraocular pressure (diopter)	mean (SD)	14.26 (2.80)	1439 (2.88)	14.13 (2.71)	14.18 (2.74)	14.29 (2.87)	14.06 (2.59)
Distance-corrected visual acuity of better-seeing eye (logMAR)	mean (SD)	0.03(0.10)	0.02 (0.09)	0.04 (0.10)	0.06 (0.15)	0.04 (0.15)	0.07 (0.16)
Distance-corrected visual acuity of worse-seeing eye (logMAR)	mean (SD)	0.12 (0.23)	0.11 (0.23)	0.12 (0.22)	0.16 (0.29)	0.15(0.31)	0.16 (0.26)
Absolute difference in distance-corrected visual acuity of both eyes (logMAR)	mean (SD)	0.09 (0.19)	0.09 (0.20)	0.08 (0.18)	0.10 (0.23)	0.11 (0.25)	0.09 (0.19)
Number of self-reported eye diseases:							
No disease	% (n)	91.2% (11,160)	91.1% (5590)	91.4% (5570)	90.5% (2516)	90.6% (1309)	90.5% (1207)
At least one disease	% (n)	8.8% (1071)	8.9% (549	8.6% (522)	9.5% (263)	9.4% (136)	9.5% (127)
At least two diseases	% (n)	0.9% (113)	0.9% (55)	1.0% (58)	0.9% (28)	0.9% (13)	1.1% (15)
Selected self-reported eye diseases:							
Glaucoma	% (n)	2.3% (278)	2.2% (138)	2.3% (140)	2.1% (58)	1.5% (22)	2.7% (36)
Ocular hypertension	% (n)	0.19% (23)	0.18% (11)	0.20% (12)	0.14% (4)	0.14%(2)	0.15% (2)
Age-related macular degeneration (AMD)	% (n)	0.4% (53)	0.4% (25)	0.5% (28)	0.6% (17)	0.6% (8)	0.7% (9)
Cataract	% (n)	0.59% (72)	0.46% (28)	0.72% (44)	0.90% (25)	0.55% (8)	1.27% (17)
Unilateral pseudophakia	% (n)	1.6% (202)	1.5% (95)	1.6% (107)	1.6% (27)	1.7% (17)	1.3% (10)
Bilateral pseudophakia	% (n)	3.1% (405)	3.0% (190)	3.3% (215)	2.7% (47)	2.5% (25)	2.9% (22)
Diabetic retinopathy	% (n)	0.11% (13)	0.15% (9)	0.07% (4)	0.14% (4)	0.14% (2)	0.15% (2)
Strabism	% (n)	2.70% (330)	2.75% (169)	2.64% (161)	2.34% (65)	2.08% (30)	2.62% (35)
Color deficiency	% (n)	0.18% (22)	0.34% (21)	0.02% (1)	0.07% (2)	0.14% (2)	0

The LFVFS showed a clear age dependency with an average of 92.8 for men and 90.5 for women in the youngest age group and 85.7 and 83.4 in the oldest age group, respectively (Tables 2 and 3). The same tendency was observed in the LFSES with 98.3 for men and 98.1 for women in the youngest age decade and 94.7 and 94.5 in the oldest age group. The largest differences between age groups were observed in both scores between the youngest group (35–44 years) and the group of 45–54 years (LFVFS approx. 5 points in both men and women, LFSES approx. 2 points difference in both men and women). Overall, men tended to have higher scores in the LFVFS (approx. 2 points), but not in the LFSES. Similar numbers were observed in the dataset restricted to participants without self-reported diseases. The short form scales showed up to 3 point lower scores compared to the long-form scales. For all scales, percentiles (in 5% steps), minimum and maximum values, stratified by sex and age decade, are presented in Additional file 1: Tables S1a–S2d. Furthermore, we present these data restricted to participants without self-reported eye diseases (Additional file 1: Tables S3a–S4d), that are very similar to the data of the total study cohort. In Table 4, we present the frequency of self-reported eye diseases and pseudophakia in the different age groups [31].

**Table 2:** NEI VFQ-25 scores in male participants of the German population-based Gutenberg Health Study (GHS), 2007–2012

	n	LFVFS		SFVFS		LFSES		SFSES	
Age decade		Mean	SD	Mean	SD	Mean	SD	Mean	SD
35–44	1304	92.8	7.7	91.5	8.6	98.3	5.1	98.0	5.9
45–54	1609	87.1	9.4	84.6	10.9	96.4	6.6	95.6	7.7
55–64	1639	86.3	10.2	84.0	11.3	95.3	8.1	94.5	9.1
65–74	1577	85.7	10.3	83.5	11.2	94.7	8.2	93.5	9.7

Scores calculated only if all items available. *LFVFS* long-form visual function scale, *SFVFS* short-form visual function scale, *LFSES* long-form socio-emotional scale, *SFSES* short-form socio-emotional scale, *SD* standard deviation

**Table 3:** NEI VFQ-25 scores in female participants of the German population-based Gutenberg Health Study (GHS), 2007–2012

	n	LFVFS		SFVFS		LFSES		SFSES	
Age decade		Mean	SD	Mean	SD	Mean	SD	Mean	SD
35–44	1439	90.5	8.5	88.9	9.5	98.1	5.1	97.7	5.9
45–54	1656	84.3	11.2	81.5	12.6	95.9	7.3	95.2	8.2
55–64	1590	84.4	11.5	82.0	12.6	95.7	7.8	94.9	9.0
65–74	1407	83.4	12.3	81.2	13.2	94.5	9.6	93.6	10.7

Scores calculated only if all items available. *LFVFS* long-form visual functioning scale, *SFVFS* short-form visual functioning scale, *LFSES* long-form socio-emotional scale, *SFSES* short-form socio-emotional scale, *SD* standard deviation

**Table 4:** Frequency of eye diseases in specific age groups of the German population-based Gutenberg Health Study (GHS), 2007–2012

	age decade	NEI VFQ-25 scales (LFVFS, SFVFS, LFSES, SFSES) available (81.5%)			NEI VFQ-25 missing, or at least one scale (LFVFS, SFVFS, LFSES, SFSES) missing (18.5%)		
		all % (n)	men % (n)	women % (n)	all% (n)	men % (n)	women%(n)
Self-reported glaucoma	35–44	0.4% (11)	0.4% (5)	0.4% (6)	0.5% (2)	0.5% (1)	0.5% (1)
	45–54	1.0% (34)	1.3% (22)	0.7%(12)	0.6% (3)	0% (0)	1.5% (3)
	55–64	2.4% (84)	2.7% (46)	2.2% (38)	1.6% (7)	1.2% (3)	2.2% (4)
	65–74	5.4% (177)	4.3% (73)	6.6% (104)	4.4% (18)	4.5% (10)	4.2% (8)
Self-reported ocular hypertension	35–44	0.1% (2)	0% (0)	0.1% (2)	0% (0)	0% (0)	0% (0)
	45–54	0.2% (6)	0.1% (2)	0.2% (4)	0% (0)	0% (0)	0% (0)
	55–64	0.2% (7)	0.1% (2)	0.3% (5)	0% (0)	0% (0)	0% (0)
	65–74	0.3% (11)	0.5% (9)	0.1% (2)	0.2% (1)	0% (0)	0.5% (1)
Self-reported age-related macular degeneration (AMD)	35–44	0% (0)	0% (0)	0% (0)	0% (0)	0% (0)	0% (0)
	45–54	0.1% (5)	0.2% (3)	0.1% (2)	0% (0)	0% (0)	0% (0)
	55–64	0.4% (13)	0.3% (6)	0.4% (7)	0.5% (2)	0.4% (1)	0.5% (1)
	65–74	1.3% (42)	1.1% (19)	1.5% (23)	2.0% (8)	1.8% (4)	2.1% (4)
Self-reported cataract	35–44	0.3% (8)	0.4% (5)	0.2% (3)	0% (0)	0% (0)	0% (0)
	45–54	0.3% (10)	0.2% (3)	0.4% (7)	0.2% (1)	0% (0)	0.5% (1)
	55–64	0.6% (22)	0.5% (8)	0.8% (14)	0.5% (2)	0.5% (1)	0.5% (1)
	65–74	1.4% (45)	1.0% (16)	1.8% (29)	2.2% (9)	1.4% (3)	3.2% (6)
Unilateral Pseudophakia	35–44	0.1% (3)	0.2% (3)	0% (0)	0.2% (1)	0.5% (1)	0% (0)
	45–54	0.4% (15)	0.6% (10)	0.3% (5)	0.6% (3)	1.0% (3)	0% (0)
	55–64	1.5% (52)	1.5% (26)	1.5% (26)	1.1% (5)	1.2% (3)	1.1% (2)
	65–74	4.1% (132)	3.3% (56)	4.8% (76)	4.4% (18)	4.5% (10)	4.2% (8)
Bilateral Pseudophakia	35–44	0.1% (2)	0.1% (2)	0% (0)	0.2% (1)	0% (0)	0.5% (1)
	45–54	0.5% (16)	0.4% (7)	0.5% (9)	0.2% (1)	0% (0)	0.5% (1)
	55–64	2.3% (80)	2.0% (35)	2.6% (45)	2.1% (9)	1.6% (4)	2.7% (5)
	65–74	9.4% (307)	8.7% (146)	10.3% (161)	8.8% (36)	9.5% (21)	7.9% (15)
Self-reported diabetic retinopathy	35–44	0% (0)	0% (0)	0% (0)	0% (0)	0% (0)	0% (0)
	45–54	0% (0)	0% (0)	0% (0)	0% (0)	0% (0)	0% (0)
	55–64	0.2% (7)	0.2% (3)	0.2% (4)	0% (0)	0% (0)	0% (0)
	65–74	0.2% (7)	0.4% (6)	0.1% (1)	0.7% (3)	0.9% (2)	0.5% (1)
Self-reported strabism	35–44	3.8% (106)	4.0% (54)	3.5% (52)	2.2% (9)	2.8% (6)	1.6% (3)
	45–54	3.1% (105)	3.0% (51)	3.1% (54)	2.0% (10)	2.7% (8)	1.0% (2)
	55–64	2.6% (89)	2.3% (40)	2.8% (49)	1.8% (8)	1.2% (3)	2.7% (5)
	65–74	1.9% (61)	1.9% (32)	1.8% (29)	1.7% (7)	2.3% (5)	1.1% (2)
Self-reported color deficiency	35–44	0.3% (9)	0.7% (9)	0% (0)	0% (0)	0% (0)	0% (0)
	45–54	0.1% (4)	0.2% (3)	0.1% (1)	0% (0)	0% (0)	0% (0)
	55–64	0.3% (9)	0.5% (9)	0% (0)	0% (0)	0% (0)	0% (0)
	65–74	0.1% (2)	0.1% (2)	0% (0)	0% (0)	0% (0)	0% (0)

In the regression analysis, female participants had on average a 1.57 lower score in the LFVFS, assuming that the other factors (age, SES, and visual acuity) were constant (Table 5). Assuming linear relationships, per five-year increase in age, the LFVFS would decrease by 0.42. Per 0.1 logMAR increase in distance-corrected visual acuity of the better-seeing eye, the LFVFS was reduced by 2.92. The absolute difference in the distance-corrected visual acuity of both eyes was associated with a decrease of 0.87 per 0.1 logMAR increase. Per 10-point increase in SES, the composite score increased by 1. In the LFSES, the influence of sex seems to be minor. Female participants had on average a 0.24 points higher score, but the statistical evidence was low (*p* = 0.073). All other factors also had a weak association with LFSES, compared to LFVFS: Per 5 year increase in age, the LFSES would decrease by 0.22. Per 0.1 logMAR increase in the better eye, the LFSES would decrease by 1.65. The difference of both eyes was associated with a decrease of 0.56 points per 0.1 logMAR increase.

**Table 5:** Linear regression results of the NEI VFQ-25 scores in the German population-based Gutenberg Health Study (GHS), 2007–2012

	LFVFS		SFVFS		LFSES		SFSES	
	Estimate [CI]	*p* value	Estimate [CI]	*p* value	Estimate [CI]	*p* value	Estimate [CI]	*p* value
Female sex	−1.57 [−1.99; −1.22]	<0.001	−1.72 [−2.12; −1.32]	<0.001	0.24 [−0.02; 0.49]	0.073	0.20 [0.43; 0.64]	0.025
Age (per 5 years)	−0.42 [−0.5; −0.34]	<0.001	−0.48 [−0.58; −0.38]	<0.001	−0.22 [−0.28; −0.15]	<0.001	−0.31 [−0.38; −0.24]	<0.001
Socio-economic status	0.11 [0.68; 0.15]	<0.001	0.12 [0.08; 0.17]	<0.001	0.08 [0.04; 0.11]	<0.001	0.08 [0.04; 0.11]	<0.001
Distance-corrected visual acuity of better-seeing eye (per 0.1 increase of logMAR)	−2.92 [−3.12; −2.72]	<0.001	−3.08 [−3.30; −2.86]	<0.001	−1.65 [−1.79; −1.50]	<0.001	−1.73 [−1.90; −1.57]	<0.001
Absolute difference in distance-corrected visual acuity of both eyes (per 0.1 increase of logMAR)	−0.87 [−0.96; −0.78]	<0.001	−0.93 [−1.04; −0.83]	<0.001	−0.56 [−0.63; −0.50]	<0.001	−0.59 [−0.67; −0.52]	<0.001
Model fit (R^2^)	0.16		0.15		0.10		0.09	<0.001

*CI* 95% confidence interval, *LFVFS* long-form visual functioning scale, *SFVFS* short-form visual functioning scale, *LFSES* long-form socio-emotional scale, *SFSES* short-form socio-emotional scale

## Discussion

Patient-reported outcomes such as QoL are important measurements to estimate the burden of diseases and are gaining importance in the evaluation of therapeutic interventions. Here, we present the distribution of vision-related quality of life, as measured by the NEI VFQ-25 after Rasch-transformation, in a large population-based German sample. Previous papers reported that unidimensionality and interval-level measurement, two of the most important requirements of such an instrument, are not met by the initially proposed NEI VFQ-25 scoring, but reference data on alternative scoring methods are lacking. To our knowledge, this is the first study presenting reference data from a large population-based study presenting data of two unidimensional scores derived from the NEI-VFQ-25. We show a clear age dependency with lower NEI VFQ-25 scores at higher ages, with the largest drop observed between the youngest age group (35–44 years) and the age group of 45–54 years. Men had higher scores than women. Lower score in LFVFS was also associated with lower visual acuity of the better-seeing eye as well as with the absolute difference in visual acuity between both eyes. These results are similar in all GHS participants as well as restricted to participants who reported having no eye diseases (Additional file 1).

The questionnaire was initially developed to capture the impact of several eye diseases on quality of life. Thus, it is not surprising that most of the scores show strong ceiling effects in our population-based sample (Additional file 1).

We provide strong evidence for associations of age and sex with LFVFS. Therefore, it is reasonable to provide reference data specific for strata defined by sex and age decades. A recent study in a German healthy working population, although using the original scores and subscores of the NEI VFQ-25, also reported a clear age dependency, with an average reduction of the NEI VFQ-25 composite score by 1 point for every decade, which is similar to our estimate (−0.6 points per 10 years) [21], but did not report on the association of vision-related QoL and sex. Sex and gender differences in QoL were not evaluated in the studies used to develop the NEI VFQ-25, but have been reported before by other studies using this questionnaire [32, 33]. There is solid evidence from other health-related QoL measurements, such as the SF-36, that women tend to report a lower health-related QoL [34, 35]. For example, data from the German National Health Interview and Examination Survey for Children and Adolescents (KiGGS) suggest that this gap begins as early as puberty: in children aged 11–13 years, mean scores in the KINDL-R tool are 75.7 for boys and 74.5 for girls; in the age group 14–17, the difference is larger (73.6 vs. 69.4) [36]. Interestingly, in our sample women had lower scores in the LFVFS, but not in the LFSES, which is less associated with visual acuity.

To the best of our knowledge, this is the largest study to report NEI VFQ-25 scores for a population-based cohort and the first to report age- and sex-specific reference data for scales based on Rasch-transformed scores. We focused not only on the visual acuity of the better-seeing eye but also took into account both eyes. Due to the strong correlation between the fellow eyes, we decided not to simply include both eyes in the model but to additionally include the absolute difference in visual acuity between both eyes. In addition to the visual acuity of the better eye, the difference was associated with a smaller but still considerable reduction in the visual function scale score (−0.89 per 0.1 difference in logMAR). This underscores the importance of both eyes for vision-related QoL, which supports recent evidence that the worse-seeing eye might have a stronger impact on VRQoL than previously assumed [37, 38]. The NEI VFQ-25 developers described only small differences in the associations of visual acuity of the worse- and better-seeing eye, but they might have missed effects because of their small sample size, the strong correlation between both eyes and because people with distinct differences between the visual acuity of both eyes might not have been included at all in their study [5].

### Strengths and limitations

The strengths of our study are the standardized study design and quality control, the broad assessment of phenotype information, the large sample size, and the population-based sampling. Since the GHS baseline recruitment was limited to participants below the age of 75, we are not able to provide data on older age groups where the prevalence of eye diseases like AMD and glaucoma is significantly higher than in younger age groups. Individuals with visual impairment are likely to be underrepresented in the GHS cohort because of their lower likelihood to participate. Hirneiss et al. found a frequency of 17% of participants with an ophthalmological disease in their sample of a working-age population in Germany, which is twice the frequency of self-reported eye diseases in our sample [21]. This does not necessarily indicate that participants with eye diseases are dramatically underrepresented in the GHS, but it might reflect that many of our participants are unaware of early asymptomatic forms of eye diseases. For example, when grading fundus images of the first 5000 GHS participants for age-related macular degeneration (AMD), we detected signs of early AMD in 11.9%, which is much higher than the self-reported prevalence of 0.4% [20, 39]. Apart from the potential underrepresentation of participants with eye diseases, we had a considerable share of participants who did not complete the NEI VFQ-25. At the beginning of the GHS, participants were asked to complete the questionnaire at home and send it back to the study center, which turned out not to work very well. Subsequently, the procedure was changed and the participants were asked to complete the questionnaire at the study center. Participants with missing NEI VFQ-25 tended to have reduced physical health, but this seems to be mainly related to general health-related risk factors and comorbidities and less related to self-reported eye diseases and ocular parameters. Therefore, we assume that the bias due to missing information on VRQoL is low. Another limitation might be that the data we used to map raw NEI VFQ data to Rasch-transformed data was collected in Australia, and that cultural differences in perceiving health-related limitations in everyday life might introduce bias. On the other hand, the traditional NEI VFQ-25 has been validated in different cultural settings with only minimal adaptions needed.

Even though the estimates from the linear regression are supported by the descriptive analyses (influence of sex and age), the results should be interpreted with care. The best R^2^ value of the regression models is 0.16 (LFVFS), meaning that only 16% of the visual functioning scale variation could be explained by the model.

## Conclusion

In summary, we report age- and sex-specific VRQoL of two unidimensional scales based on Rasch-transformed NEI VFQ-25 questionnaire data from a large population-based German cohort that could be used as a reference for comparison in future studies. Furthermore, we show a clear age dependency for VRQoL, with lower scores at higher ages. The largest drop in VRQoL was observed between the age group of 35–44 years and the age group of 45–54 years. Worse VRQoL was associated with the visual acuity of the better-seeing eye and additionally with the difference between both eyes.

## Additional file

Additional file 1: Supplementary Tables.
**Table S1a.** NEI VFQ-25 scores in men aged 35–44 years of the German population-based Gutenberg Health Study (GHS), 2007–2012. **Table S1b.** NEI VFQ-25 scores in men aged 45–54 years of the German population-based Gutenberg Health Study (GHS), 2007–2012. **Table S1c.** NEI VFQ-25 scores in men aged 55–64 years of the German population-based Gutenberg Health Study (GHS), 2007–2012. **Table S1d.** NEI VFQ-25 scores in men aged 65–74 years of the German population-based Gutenberg Health Study (GHS), 2007–2012. **Table S2a.** NEI VFQ-25 scores in women aged 35–44 years of the German population-based Gutenberg Health Study (GHS), 2007–2012. **Table S2b.** NEI VFQ-25 scores in women aged 45–54 years of the German population-based Gutenberg Health Study (GHS), 2007–2012. **Table S2c.** NEI VFQ-25 scores in women aged 55–64 years of the German population-based Gutenberg Health Study (GHS), 2007–2012. **Table S2d.** NEI VFQ-25 in women aged 65–74 years of the German population-based Gutenberg Health Study (GHS), 2007–2012. **Table S3a.** NEI VFQ-25 in men without self-reported eye diseases aged 35–44 years of the German population-based Gutenberg Health Study (GHS), 2007–2012. **Table S3b.** NEI VFQ-25 in men without self-reported eye diseases aged 45–54 years of the German population-based Gutenberg Health Study (GHS), 2007–2012. **Table S3c.** NEI VFQ-25 scores in men without self-reported eye diseases aged 55–64 years of the German population-based Gutenberg Health Study (GHS), 2007–2012. **Table S3d.** NEI VFQ-25 scores in men without self-reported eye diseases aged 65–74 years of the German population-based Gutenberg Health Study (GHS), 2007–2012. **Table S4a.** NEI VFQ-25 scores in women without self-reported eye diseases aged 35–44 years of the German population-based Gutenberg Health Study (GHS), 2007–2012. **Table S4b.** NEI VFQ-25 scores in women without self-reported eye diseases aged 45–54 years of the German population-based Gutenberg Health Study (GHS), 2007–2012. **Table S4c.** NEI VFQ-25 scores in women without self-reported eye diseases aged 55–64 years of the German population-based Gutenberg Health Study (GHS), 2007–2012. **Table S4d.** NEI VFQ-25 scores in women without self-reported eye diseases aged 65–74 years of the German population-based Gutenberg Health Study (GHS), 2007–2012. (DOC 461 kb)

## Abbreviations

CI: Confidence interval
GHS: Gutenberg Health Study
LFSES: Long-form socio-emotional scale
LFVFS: Long-form visual functioning scale
NEI VFQ-25: National Eye Institute 25-Item Visual Function Questionnaire
QoL: Quality of life
SES: Socioeconomic status
SFSES: Short-form socio-emotional scale
SFVFS: Short-form visual functioning scale
VRQoL: Vision-related QoL
