# Supplementary material for: The National Eye Institute 25-Item Visual Function Questionnaire (NEI VFQ-25) – reference data from the German population-based Gutenberg Health Study (GHS)
Source: Health Qual Life Outcomes. 2017 Aug 8;15:156. doi: 10.1186/s12955-017-0732-7 (PMC5549396; doi:10.1186/s12955-017-0732-7)
Supplement: Additional file 1: Supplementary Tables. — Table S1a. NEI VFQ-25 scores in men aged 35–44 years of the German population-based Gutenberg Health Study (GHS), 2007–2012. Table S1b. NEI VFQ-25 scores in men aged 45–54 years of the German population-based Gutenberg Health Study (GHS), 2007–2012. Table S1c. NEI VFQ-25 scores in men aged 55–64 years of the German population-based Gutenberg Health Study (GHS), 2007–2012. Table S1d. NEI VFQ-25 scores in men aged 65–74 years of the German population-based Gutenberg Health Study (GHS), 2007–2012. Table S2a. NEI VFQ-25 scores in women aged 35–44 years of the German population-based Gutenberg Health Study (GHS), 2007–2012. Table S2b. NEI VFQ-25 scores in women aged 45–54 years of the German population-based Gutenberg Health Study (GHS), 2007–2012. Table S2c. NEI VFQ-25 scores in women aged 55–64 years of the German population-based Gutenberg Health Study (GHS), 2007–2012. Table S2d. NEI VFQ-25 in women aged 65–74 years of the German population-based Gutenberg Health Study (GHS), 2007–2012. Table S3a. NEI VFQ-25 in men without self-reported eye diseases aged 35–44 years of the German population-based Gutenberg Health Study (GHS), 2007–2012. Table S3b. NEI VFQ-25 in men without self-reported eye diseases aged 45–54 years of the German population-based Gutenberg Health Study (GHS), 2007–2012. Table S3c. NEI VFQ-25 scores in men without self-reported eye diseases aged 55–64 years of the German population-based Gutenberg Health Study (GHS), 2007–2012. Table S3d. NEI VFQ-25 scores in men without self-reported eye diseases aged 65–74 years of the German population-based Gutenberg Health Study (GHS), 2007–2012. Table S4a. NEI VFQ-25 scores in women without self-reported eye diseases aged 35–44 years of the German population-based Gutenberg Health Study (GHS), 2007–2012. Table S4b. NEI VFQ-25 scores in women without self-reported eye diseases aged 45–54 years of the German population-based Gutenberg Health Study (GHS), 2007–2012. Table S4c. NEI VFQ-25 scores in women without sel [file 12955_2017_732_MOESM1_ESM.doc]

Additional file 1

Table S1a: NEI VFQ-25 scores in men aged 35-44 years of the German population-based Gutenberg Health Study (GHS), 2007-2012

|  | | **LFVFS** | **SFVFS** | **LFSES** | **SFSES** |
| --- | --- | --- | --- | --- | --- |
| **N** | | 1304 | 1304 | 1304 | 1304 |
| **Mean** | | 92.8 | 91.5 | 98.3 | 98.0 |
| **SD** | | 7.7 | 8.6 | 5.1 | 5.9 |
| **Minimum** | | 21.7 | 23.1 | 46.1 | 42.1 |
| **Maximum** | | 100 | 100 | 100 | 100 |
| **Percentiles** | **5** | 78.5 | 75.7 | 91.2 | 87.8 |
|  | **10** | 84.0 | 79.4 | 94.6 | 95.4 |
|  | **15** | 86.8 | 83.0 | 97.0 | 95.7 |
|  | **20** | 89.5 | 86.5 | 97.5 | 100 |
|  | **25** | 89.6 | 90.1 | 100 | 100 |
|  | **30** | 92.3 | 90.1 | 100 | 100 |
|  | **35** | 92.3 | 90.1 | 100 | 100 |
|  | **40** | 92.3 | 92.8 | 100 | 100 |
|  | **45** | 95.1 | 93.7 | 100 | 100 |
|  | **50** | 95.1 | 93.7 | 100 | 100 |
|  | **55** | 95.1 | 93.7 | 100 | 100 |
|  | **60** | 95.1 | 93.7 | 100 | 100 |
|  | **65** | 95.1 | 93.7 | 100 | 100 |
|  | **70** | 97.2 | 96.4 | 100 | 100 |
|  | **75** | 100 | 100 | 100 | 100 |
|  | **80** | 100 | 100 | 100 | 100 |
|  | **85** | 100 | 100 | 100 | 100 |
|  | **90** | 100 | 100 | 100 | 100 |
|  | **95** | 100 | 100 | 100 | 100 |

LFVFS: long-form visual functioning scale; SFVFS: short-form visual functioning scale; LFSES: long-form socio-emotional scale; SFSES: short-form socio-emotional scale.

Table S1b: NEI VFQ-25 scores in men aged 45-54 years of the German population-based Gutenberg Health Study (GHS), 2007-2012

|  | | **LFVFS** | **SFVFS** | **LFSES** | **SFSES** |
| --- | --- | --- | --- | --- | --- |
| **N** | | 1619 | 1619 | 1619 | 1619 |
| **Mean** | | 87.1 | 84.6 | 96.4 | 95.6 |
| **SD** | | 9.4 | 10.9 | 6.6 | 7.7 |
| **Minimum** | | 43.8 | 34.1 | 49.0 | 38.3 |
| **Maximum** | | 100 | 100 | 100 | 100 |
| **Percentiles** | **5** | 67.9 | 65.0 | 83.1 | 79.4 |
|  | **10** | 75.0 | 68.6 | 89.2 | 87.1 |
|  | **15** | 78.5 | 72.3 | 91.7 | 91.3 |
|  | **20** | 80.5 | 75.7 | 94.1 | 91.4 |
|  | **25** | 81.4 | 79.3 | 94.6 | 95.6 |
|  | **30** | 84.0 | 79.4 | 97.0 | 95.7 |
|  | **35** | 84.2 | 82.9 | 97.0 | 95.7 |
|  | **40** | 86.8 | 83.0 | 97.1 | 95.7 |
|  | **45** | 87.0 | 86.5 | 100 | 100 |
|  | **50** | 89.6 | 86.5 | 100 | 100 |
|  | **55** | 89.6 | 86.5 | 100 | 100 |
|  | **60** | 92.3 | 90.1 | 100 | 100 |
|  | **65** | 92.3 | 90.1 | 100 | 100 |
|  | **70** | 92.3 | 90.1 | 100 | 100 |
|  | **75** | 95.1 | 93.7 | 100 | 100 |
|  | **80** | 95.1 | 93.7 | 100 | 100 |
|  | **85** | 95.1 | 93.7 | 100 | 100 |
|  | **90** | 97.2 | 96.4 | 100 | 100 |
|  | **95** | 100 | 100 | 100 | 100 |

LFVFS: long-form visual functioning scale; SFVFS: short-form visual functioning scale; LFSES: long-form socio-emotional scale; SFSES: short-form socio-emotional scale.

Table S1c: NEI VFQ-25 scores in men aged 55-64 years of the German population-based Gutenberg Health Study (GHS), 2007-2012

|  | | **LFVFS** | **SFVFS** | **LFSES** | **SFSES** |
| --- | --- | --- | --- | --- | --- |
| **N** | | 1639 | 1639 | 1639 | 1639 |
| **Mean** | | 86.3 | 84.0 | 95.3 | 94.5 |
| **SD** | | 10.2 | 11.3 | 8.1 | 9.1 |
| **Minimum** | | 29.2 | 19.0 | 26.2 | 26.7 |
| **Maximum** | | 100 | 100 | 100 | 100 |
| **Percentiles** | **5** | 67.0 | 64.1 | 77.4 | 74.9 |
|  | **10** | 72.2 | 68.5 | 85.3 | 82.6 |
|  | **15** | 75.7 | 72.1 | 89.6 | 87.1 |
|  | **20** | 78.5 | 75.7 | 91.6 | 91.3 |
|  | **25** | 81.3 | 78.5 | 94.1 | 91.4 |
|  | **30** | 81.5 | 79.4 | 94.6 | 95.6 |
|  | **35** | 84.1 | 82.9 | 97.0 | 95.7 |
|  | **40** | 86.8 | 83.0 | 97.0 | 95.7 |
|  | **45** | 86.8 | 86.4 | 97.5 | 100 |
|  | **50** | 89.6 | 86.5 | 100 | 100 |
|  | **55** | 89.6 | 86.5 | 100 | 100 |
|  | **60** | 92.3 | 90.1 | 100 | 100 |
|  | **65** | 92.3 | 90.1 | 100 | 100 |
|  | **70** | 92.3 | 90.1 | 100 | 100 |
|  | **75** | 95.1 | 93.7 | 100 | 100 |
|  | **80** | 95.1 | 93.7 | 100 | 100 |
|  | **85** | 95.1 | 93.7 | 100 | 100 |
|  | **90** | 95.1 | 93.7 | 100 | 100 |
|  | **95** | 100 | 100 | 100 | 100 |

LFVFS: long-form visual functioning scale; SFVFS: short-form visual functioning scale; LFSES: long-form socio-emotional scale; SFSES: short-form socio-emotional scale.

**Table S1d: NEI VFQ-25 scores in men aged 65-74 years of the German population-based Gutenberg Health Study (GHS), 2007-2012**

|  | | **LFVFS** | **SFVFS** | **LFSES** | **SFSES** |
| --- | --- | --- | --- | --- | --- |
| **N** | | 1577 | 1577 | 1577 | 1577 |
| **Mean** | | 85.7 | 83.5 | 94.7 | 93.5 |
| **SD** | | 10.3 | 11.2 | 8.2 | 9.7 |
| **Minimum** | | 21.6 | 23.1 | 34.0 | 32.9 |
| **Maximum** | | 100 | 100 | 100 | 100 |
| **Percentiles** | **5** | 66.7 | 62.9 | 76.5 | 71.5 |
|  | **10** | 71.4 | 68.5 | 82.9 | 79.6 |
|  | **15** | 75.7 | 72.1 | 88.2 | 84.2 |
|  | **20** | 78.5 | 75.7 | 91.2 | 87.7 |
|  | **25** | 78.7 | 75.8 | 92.6 | 91.3 |
|  | **30** | 81.3 | 79.3 | 94.1 | 91.4 |
|  | **35** | 84.0 | 79.4 | 94.6 | 95.6 |
|  | **40** | 84.2 | 82.9 | 97.0 | 95.6 |
|  | **45** | 86.8 | 83.0 | 97.0 | 95.7 |
|  | **50** | 88.1 | 86.5 | 97.5 | 100 |
|  | **55** | 89.6 | 86.5 | 100 | 100 |
|  | **60** | 89.6 | 90.1 | 100 | 100 |
|  | **65** | 92.3 | 90.1 | 100 | 100 |
|  | **70** | 92.3 | 90.1 | 100 | 100 |
|  | **75** | 93.4 | 93.7 | 100 | 100 |
|  | **80** | 95.1 | 93.7 | 100 | 100 |
|  | **85** | 95.1 | 93.7 | 100 | 100 |
|  | **90** | 95.1 | 93.7 | 100 | 100 |
|  | **95** | 100 | 100 | 100 | 100 |

LFVFS: long-form visual functioning scale; SFVFS: short-form visual functioning scale; LFSES: long-form socio-emotional scale; SFSES: short-form socio-emotional scale.

Table S2a: NEI VFQ-25 scores in women aged 35-44 years of the German population-based Gutenberg Health Study (GHS), 2007-2012

|  | | **LFVFS** | **SFVFS** | **LFSES** | **SFSES** |
| --- | --- | --- | --- | --- | --- |
| **N** | | 1439 | 1439 | 1439 | 1439 |
| **Mean** | | 90.5 | 88.9 | 98.1 | 97.7 |
| **SD** | | 8.5 | 9.5 | 5.1 | 5.9 |
| **Minimum** | | 33.0 | 34.0 | 51.8 | 48.9 |
| **Maximum** | | 100 | 100 | 100 | 100 |
| **Percentiles** | **5** | 73.4 | 71.3 | 89.2 | 87.1 |
|  | **10** | 78.6 | 75.7 | 94.1 | 91.4 |
|  | **15** | 81.4 | 79.3 | 97.0 | 95.6 |
|  | **20** | 84.1 | 82.9 | 97.0 | 95.7 |
|  | **25** | 86.8 | 83.0 | 100 | 100 |
|  | **30** | 87.0 | 86.5 | 100 | 100 |
|  | **35** | 89.6 | 86.5 | 100 | 100 |
|  | **40** | 89.6 | 90.1 | 100 | 100 |
|  | **45** | 92.3 | 90.1 | 100 | 100 |
|  | **50** | 92.3 | 90.1 | 100 | 100 |
|  | **55** | 92.3 | 90.1 | 100 | 100 |
|  | **60** | 95.1 | 93.7 | 100 | 100 |
|  | **65** | 95.1 | 93.7 | 100 | 100 |
|  | **70** | 95.1 | 93.7 | 100 | 100 |
|  | **75** | 95.1 | 93.7 | 100 | 100 |
|  | **80** | 97.2 | 96.4 | 100 | 100 |
|  | **85** | 100 | 100 | 100 | 100 |
|  | **90** | 100 | 100 | 100 | 100 |
|  | **95** | 100 | 100 | 100 | 100 |

LFVFS: long-form visual functioning scale; SFVFS: short-form visual functioning scale; LFSES: long-form socio-emotional scale; SFSES: short-form socio-emotional scale.

**Table S2b: NEI VFQ-25 scores in women aged 45-54 years of the German population-based Gutenberg Health Study (GHS), 2007-2012**

|  | | **LFVFS** | **SFVFS** | **LFSES** | **SFSES** |
| --- | --- | --- | --- | --- | --- |
| **N** | | 1656 | 1656 | 1656 | 1656 |
| **Mean** | | 84.3 | 81.5 | 95.9 | 95.2 |
| **SD** | | 11.2 | 12.6 | 7.3 | 8.2 |
| **Minimum** | | 6.0 | 7.5 | 39.9 | 37.3 |
| **Maximum** | | 100 | 100 | 100 | 100 |
| **Percentiles** | **5** | 64.2 | 57.8 | 82.2 | 78.3 |
|  | **10** | 70.2 | 64.9 | 88.2 | 84.2 |
|  | **15** | 73.1 | 68.5 | 91.1 | 87.8 |
|  | **20** | 75.9 | 72.1 | 92.6 | 91.3 |
|  | **25** | 78.5 | 75.7 | 94.1 | 92.1 |
|  | **30** | 81.3 | 75.8 | 95.1 | 95.6 |
|  | **35** | 81.4 | 79.3 | 97.0 | 95.7 |
|  | **40** | 84.0 | 79.4 | 97.1 | 95.7 |
|  | **45** | 84.2 | 82.9 | 97.5 | 100 |
|  | **50** | 86.8 | 83.0 | 100 | 100 |
|  | **55** | 87.0 | 86.5 | 100 | 100 |
|  | **60** | 89.6 | 86.5 | 100 | 100 |
|  | **65** | 89.6 | 86.6 | 100 | 100 |
|  | **70** | 92.3 | 90.1 | 100 | 100 |
|  | **75** | 92.3 | 90.1 | 100 | 100 |
|  | **80** | 92.3 | 92.8 | 100 | 100 |
|  | **85** | 95.1 | 93.7 | 100 | 100 |
|  | **90** | 95.1 | 93.7 | 100 | 100 |
|  | **95** | 100 | 100 | 100 | 100 |

LFVFS: long-form visual functioning scale; SFVFS: short-form visual functioning scale; LFSES: long-form socio-emotional scale; SFSES: short-form socio-emotional scale.

**Table S2c: NEI VFQ-25 scores in women aged 55-64 years of the German population-based Gutenberg Health Study (GHS), 2007-2012**

|  | | **LFVFS** | **SFVFS** | **LFSES** | **SFSES** |
| --- | --- | --- | --- | --- | --- |
| **N** | | 1590 | 1590 | 1590 | 1590 |
| **Mean** | | 84.4 | 82.0 | 95.7 | 94.9 |
| **SD** | | 11.5 | 12.6 | 7.8 | 9.0 |
| **Minimum** | | 12.7 | 11.9 | 35.5 | 32.1 |
| **Maximum** | | 100 | 100 | 100 | 100 |
| **Percentiles** | **5** | 62.1 | 57.0 | 78.9 | 75.3 |
|  | **10** | 68.1 | 65.0 | 86.7 | 83.2 |
|  | **15** | 73.1 | 68.6 | 89.7 | 87.1 |
|  | **20** | 75.9 | 72.1 | 92.1 | 91.3 |
|  | **25** | 78.5 | 75.7 | 94.1 | 92.1 |
|  | **30** | 81.3 | 75.8 | 97.0 | 95.6 |
|  | **35** | 81.5 | 79.4 | 97.0 | 95.7 |
|  | **40** | 84.1 | 82.9 | 97.1 | 96.4 |
|  | **45** | 86.8 | 83.0 | 100 | 100 |
|  | **50** | 86.8 | 83.0 | 100 | 100 |
|  | **55** | 89.5 | 86.5 | 100 | 100 |
|  | **60** | 89.6 | 86.5 | 100 | 100 |
|  | **65** | 89.6 | 90.1 | 100 | 100 |
|  | **70** | 92.3 | 90.1 | 100 | 100 |
|  | **75** | 92.3 | 90.1 | 100 | 100 |
|  | **80** | 95.1 | 93.7 | 100 | 100 |
|  | **85** | 95.1 | 93.7 | 100 | 100 |
|  | **90** | 95.1 | 93.7 | 100 | 100 |
|  | **95** | 100 | 100 | 100 | 100 |

LFVFS: long-form visual functioning scale; SFVFS: short-form visual functioning scale; LFSES: long-form socio-emotional scale; SFSES: short-form socio-emotional scale.

**Table S2d: NEI VFQ-25 scores in women aged 65-74 years of the German population-based Gutenberg Health Study (GHS), 2007-2012**

|  | | **LFVFS** | **SFVFS** | **LFSES** | **SFSES** |
| --- | --- | --- | --- | --- | --- |
| **N** | | 1407 | 1407 | 1407 | 1407 |
| **Mean** | | 83.4 | 81.2 | 94.5 | 93.6 |
| **SD** | | 12.3 | 13.2 | 9.6 | 10.7 |
| **Minimum** | | 3.7 | 4.4 | 28.6 | 24.6 |
| **Maximum** | | 100 | 100 | 100 | 100 |
| **Percentiles** | **5** | 59.8 | 57.0 | 74.0 | 68.9 |
|  | **10** | 67.5 | 64.1 | 82.8 | 79.0 |
|  | **15** | 70.4 | 68.4 | 87.7 | 84.2 |
|  | **20** | 73.3 | 71.3 | 91.2 | 88.2 |
|  | **25** | 75.9 | 74.8 | 94.1 | 91.3 |
|  | **30** | 78.5 | 75.7 | 94.1 | 92.8 |
|  | **35** | 81.3 | 79.3 | 97.0 | 95.6 |
|  | **40** | 84.0 | 79.4 | 97.0 | 95.7 |
|  | **45** | 84.2 | 83.0 | 97.1 | 96.4 |
|  | **50** | 86.8 | 83.0 | 100 | 100 |
|  | **55** | 86.9 | 86.5 | 100 | 100 |
|  | **60** | 89.6 | 86.5 | 100 | 100 |
|  | **65** | 89.6 | 90.1 | 100 | 100 |
|  | **70** | 92.3 | 90.1 | 100 | 100 |
|  | **75** | 92.3 | 90.1 | 100 | 100 |
|  | **80** | 92.3 | 92.8 | 100 | 100 |
|  | **85** | 95.1 | 93.7 | 100 | 100 |
|  | **90** | 95.1 | 93.7 | 100 | 100 |
|  | **95** | 97.2 | 96.4 | 100 | 100 |

LFVFS: long-form visual functioning scale; SFVFS: short-form visual functioning scale; LFSES: long-form socio-emotional scale; SFSES: short-form socio-emotional scale.

**Table S3a: NEI VFQ-25 scores in men without self-reported eye diseases aged 35-44 years of the German population-based Gutenberg Health Study (GHS), 2007-2012**

|  | | **LFVFS** | **SFVFS** | **LFSES** | **SFSES** |
| --- | --- | --- | --- | --- | --- |
| **N** | | 1186 | 1186 | 1186 | 1186 |
| **Mean** | | 93.3 | 92.1 | 98.6 | 98.3 |
| **SD** | | 6.8 | 7.8 | 4.3 | 5.2 |
| **Minimum** | | 47.0 | 45.1 | 51.7 | 42.1 |
| **Maximum** | | 100 | 100 | 100 | 100 |
| **Percentiles** | **5** | 80.5 | 75.7 | 91.6 | 91.3 |
|  | **10** | 84.1 | 82.9 | 97.0 | 95.6 |
|  | **15** | 86.8 | 83.2 | 97.0 | 95.7 |
|  | **20** | 89.6 | 86.5 | 100 | 100 |
|  | **25** | 89.6 | 90.1 | 100 | 100 |
|  | **30** | 92.3 | 90.1 | 100 | 100 |
|  | **35** | 92.3 | 90.1 | 100 | 100 |
|  | **40** | 94.5 | 93.7 | 100 | 100 |
|  | **45** | 95.1 | 93.7 | 100 | 100 |
|  | **50** | 95.1 | 93.7 | 100 | 100 |
|  | **55** | 95.1 | 93.7 | 100 | 100 |
|  | **60** | 95.1 | 93.7 | 100 | 100 |
|  | **65** | 95.1 | 93.7 | 100 | 100 |
|  | **70** | 97.2 | 96.4 | 100 | 100 |
|  | **75** | 100 | 100 | 100 | 100 |
|  | **80** | 100 | 100 | 100 | 100 |
|  | **85** | 100 | 100 | 100 | 100 |
|  | **90** | 100 | 100 | 100 | 100 |
|  | **95** | 100 | 100 | 100 | 100 |

LFVFS: long-form visual functioning scale; SFVFS: short-form visual functioning scale; LFSES: long-form socio-emotional scale; SFSES: short-form socio-emotional scale.

**Table S3b: NEI VFQ-25 scores in men without self-reported eye diseases aged 45-54 years of the German population-based Gutenberg Health Study (GHS), 2007-2012**

|  | | **LFVFS** | **SFVFS** | **LFSES** | **SFSES** |
| --- | --- | --- | --- | --- | --- |
| **N** | | 1485 | 1485 | 1485 | 1485 |
| **Mean** | | 87.3 | 84.8 | 96.6 | 95.8 |
| **SD** | | 9.3 | 10.8 | 6.3 | 7.4 |
| **Minimum** | | 43.8 | 34.1 | 49.0 | 38.3 |
| **Maximum** | | 100 | 100 | 100 | 100 |
| **Percentiles** | **5** | 70.1 | 65.0 | 84.9 | 79.9 |
|  | **10** | 75.1 | 71.3 | 91.1 | 87.1 |
|  | **15** | 78.5 | 74.8 | 92.2 | 91.3 |
|  | **20** | 81.3 | 75.7 | 94.1 | 91.4 |
|  | **25** | 81.4 | 79.3 | 94.6 | 95.6 |
|  | **30** | 84.0 | 79.4 | 97.0 | 95.7 |
|  | **35** | 86.0 | 82.9 | 97.1 | 95.7 |
|  | **40** | 86.8 | 83.0 | 97.1 | 96.4 |
|  | **45** | 89.5 | 86.5 | 100 | 100 |
|  | **50** | 89.6 | 86.5 | 100 | 100 |
|  | **55** | 89.6 | 89.5 | 100 | 100 |
|  | **60** | 92.3 | 90.1 | 100 | 100 |
|  | **65** | 92.3 | 90.1 | 100 | 100 |
|  | **70** | 92.3 | 90.1 | 100 | 100 |
|  | **75** | 95.1 | 93.7 | 100 | 100 |
|  | **80** | 95.1 | 93.7 | 100 | 100 |
|  | **85** | 95.1 | 93.7 | 100 | 100 |
|  | **90** | 97.2 | 96.4 | 100 | 100 |
|  | **95** | 100 | 100 | 100 | 100 |

LFVFS: long-form visual functioning scale; SFVFS: short-form visual functioning scale; LFSES: long-form socio-emotional scale; SFSES: short-form socio-emotional scale.

**Table S3c: NEI VFQ-25 scores in men without self-reported eye diseases aged 55-64 years of the German population-based Gutenberg Health Study (GHS), 2007-2012**

|  | | **LFVFS** | **SFVFS** | **LFSES** | **SFSES** |
| --- | --- | --- | --- | --- | --- |
| **N** | | 1501 | 1501 | 1501 | 1501 |
| **Mean** | | 86.7 | 84.4 | 95.6 | 94.8 |
| **SD** | | 9.8 | 11.0 | 7.7 | 8.7 |
| **Minimum** | | 29.2 | 19.0 | 43.8 | 44.8 |
| **Maximum** | | 100 | 100 | 100 | 100 |
| **Percentiles** | **5** | 67.6 | 64.1 | 78.1 | 75.4 |
|  | **10** | 72.4 | 68.5 | 85.8 | 83.2 |
|  | **15** | 75.9 | 72.1 | 91.1 | 87.1 |
|  | **20** | 78.5 | 75.7 | 92.3 | 91.3 |
|  | **25** | 81.3 | 79.3 | 94.1 | 91.4 |
|  | **30** | 84.0 | 79.4 | 94.6 | 95.6 |
|  | **35** | 84.1 | 82.9 | 97.0 | 95.7 |
|  | **40** | 86.8 | 83.0 | 97.1 | 95.7 |
|  | **45** | 87.0 | 86.4 | 97.5 | 100 |
|  | **50** | 89.6 | 86.5 | 100 | 100 |
|  | **55** | 89.6 | 89.3 | 100 | 100 |
|  | **60** | 92.3 | 90.1 | 100 | 100 |
|  | **65** | 92.3 | 90.1 | 100 | 100 |
|  | **70** | 92.3 | 90.1 | 100 | 100 |
|  | **75** | 95.1 | 93.7 | 100 | 100 |
|  | **80** | 95.1 | 93.7 | 100 | 100 |
|  | **85** | 95.1 | 93.7 | 100 | 100 |
|  | **90** | 95.1 | 93.7 | 100 | 100 |
|  | **95** | 100 | 100 | 100 | 100 |

LFVFS: long-form visual functioning scale; SFVFS: short-form visual functioning scale; LFSES: long-form socio-emotional scale; SFSES: short-form socio-emotional scale.

**Table S3d: NEI VFQ-25 scores in men without self-reported eye diseases aged 65-74 years of the German population-based Gutenberg Health Study (GHS), 2007-2012**

|  | | **LFVFS** | **SFVFS** | **LFSES** | **SFSES** |
| --- | --- | --- | --- | --- | --- |
| **N** | | 1418 | 1418 | 1418 | 1418 |
| **Mean** | | 86.0 | 83.9 | 94.9 | 93.8 |
| **SD** | | 10.1 | 11.0 | 7.8 | 9.3 |
| **Minimum** | | 21.6 | 23.1 | 49.1 | 48.3 |
| **Maximum** | | 100 | 100 | 100 | 100 |
| **Percentiles** | **5** | 67.6 | 64.1 | 76.5 | 71.9 |
|  | **10** | 72.5 | 68.6 | 83.7 | 79.9 |
|  | **15** | 75.7 | 72.1 | 88.2 | 84.2 |
|  | **20** | 78.5 | 75.7 | 91.2 | 87.8 |
|  | **25** | 81.3 | 75.8 | 94.1 | 91.3 |
|  | **30** | 81.3 | 79.3 | 94.1 | 91.4 |
|  | **35** | 84.0 | 79.4 | 94.6 | 95.6 |
|  | **40** | 86.1 | 83.0 | 97.0 | 95.7 |
|  | **45** | 86.8 | 86.1 | 97.0 | 95.7 |
|  | **50** | 89.5 | 86.5 | 97.5 | 100 |
|  | **55** | 89.6 | 86.5 | 100 | 100 |
|  | **60** | 89.6 | 90.1 | 100 | 100 |
|  | **65** | 92.3 | 90.1 | 100 | 100 |
|  | **70** | 92.3 | 90.1 | 100 | 100 |
|  | **75** | 94.5 | 93.7 | 100 | 100 |
|  | **80** | 95.1 | 93.7 | 100 | 100 |
|  | **85** | 95.1 | 93.7 | 100 | 100 |
|  | **90** | 95.1 | 93.7 | 100 | 100 |
|  | **95** | 100 | 100 | 100 | 100 |

LFVFS: long-form visual functioning scale; SFVFS: short-form visual functioning scale; LFSES: long-form socio-emotional scale; SFSES: short-form socio-emotional scale.

**Table S4a: NEI VFQ-25 scores in women without self-reported eye diseases aged 35-44 years of the German population-based Gutenberg Health Study (GHS), 2007-2012**

|  | | **LFVFS** | **SFVFS** | **LFSES** | **SFSES** |
| --- | --- | --- | --- | --- | --- |
| **N** | | 1334 | 1334 | 1334 | 1334 |
| **Mean** | | 90.7 | 89.2 | 98.2 | 97.9 |
| **SD** | | 8.3 | 9.3 | 5.0 | 5.8 |
| **Minimum** | | 33.0 | 34.0 | 51.8 | 48.9 |
| **Maximum** | | 100 | 100 | 100 | 100 |
| **Percentiles** | **5** | 75.7 | 72.1 | 91.1 | 87.7 |
|  | **10** | 78.7 | 75.8 | 94.1 | 92.1 |
|  | **15** | 84.0 | 79.4 | 97.0 | 95.7 |
|  | **20** | 84.2 | 83.0 | 97.0 | 95.7 |
|  | **25** | 86.8 | 86.4 | 100 | 100 |
|  | **30** | 89.5 | 86.5 | 100 | 100 |
|  | **35** | 89.6 | 86.5 | 100 | 100 |
|  | **40** | 89.6 | 90.1 | 100 | 100 |
|  | **45** | 92.3 | 90.1 | 100 | 100 |
|  | **50** | 92.3 | 90.1 | 100 | 100 |
|  | **55** | 92.3 | 92.8 | 100 | 100 |
|  | **60** | 95.1 | 93.7 | 100 | 100 |
|  | **65** | 95.1 | 93.7 | 100 | 100 |
|  | **70** | 95.1 | 93.7 | 100 | 100 |
|  | **75** | 95.1 | 93.7 | 100 | 100 |
|  | **80** | 97.2 | 96.4 | 100 | 100 |
|  | **85** | 100 | 100 | 100 | 100 |
|  | **90** | 100 | 100 | 100 | 100 |
|  | **95** | 100 | 100 | 100 | 100 |

LFVFS: long-form visual functioning scale; SFVFS: short-form visual functioning scale; LFSES: long-form socio-emotional scale; SFSES: short-form socio-emotional scale.

**Table S4b: NEI VFQ-25 composite score and subscale scores in women without self-reported eye diseases aged 45-54 years of the German population-based Gutenberg Health Study (GHS), 2007-2012**

|  | | **LFVFS** | **SFVFS** | **LFSES** | **SFSES** |
| --- | --- | --- | --- | --- | --- |
| **N** | | 1525 | 1525 | 1525 | 1525 |
| **Mean** | | 84.6 | 81.8 | 96.1 | 95.4 |
| **SD** | | 10.9 | 12.3 | 6.9 | 8.0 |
| **Minimum** | | 26.8 | 26.4 | 41.5 | 37.3 |
| **Maximum** | | 100 | 100 | 100 | 100 |
| **Percentiles** | **5** | 64.5 | 57.8 | 82.5 | 79.0 |
|  | **10** | 70.3 | 65.0 | 88.3 | 86.0 |
|  | **15** | 73.2 | 68.6 | 91.2 | 88.5 |
|  | **20** | 75.9 | 72.1 | 94.1 | 91.3 |
|  | **25** | 78.5 | 75.7 | 94.1 | 92.2 |
|  | **30** | 81.3 | 75.8 | 97.0 | 95.6 |
|  | **35** | 81.4 | 79.3 | 97.0 | 95.7 |
|  | **40** | 84.0 | 79.4 | 97.1 | 96.0 |
|  | **45** | 84.2 | 83.0 | 100 | 100 |
|  | **50** | 86.8 | 83.0 | 100 | 100 |
|  | **55** | 89.0 | 86.5 | 100 | 100 |
|  | **60** | 89.6 | 86.5 | 100 | 100 |
|  | **65** | 89.6 | 89.3 | 100 | 100 |
|  | **70** | 92.3 | 90.1 | 100 | 100 |
|  | **75** | 92.3 | 90.1 | 100 | 100 |
|  | **80** | 94.5 | 92.9 | 100 | 100 |
|  | **85** | 95.1 | 93.7 | 100 | 100 |
|  | **90** | 95.1 | 93.7 | 100 | 100 |
|  | **95** | 100 | 100 | 100 | 100 |

LFVFS: long-form visual functioning scale; SFVFS: short-form visual functioning scale; LFSES: long-form socio-emotional scale; SFSES: short-form socio-emotional scale.

**Table S4c: NEI VFQ-25 composite score and subscale scores in women without self-reported eye diseases aged 55-64 years of the German population-based Gutenberg Health Study (GHS), 2007-2012**

|  | | **LFVFS** | **SFVFS** | **LFSES** | **SFSES** |
| --- | --- | --- | --- | --- | --- |
| **N** | | 1445 | 1445 | 1445 | 1445 |
| **Mean** | | 84.6 | 82.1 | 95.8 | 95.1 |
| **SD** | | 11.4 | 12.5 | 7.5 | 8.7 |
| **Minimum** | | 12.7 | 11.9 | 35.5 | 32.1 |
| **Maximum** | | 100 | 100 | 100 | 100 |
| **Percentiles** | **5** | 62.1 | 57.0 | 79.8 | 76.2 |
|  | **10** | 69.5 | 65.0 | 87.4 | 83.8 |
|  | **15** | 73.1 | 68.6 | 91.1 | 87.7 |
|  | **20** | 75.9 | 72.1 | 94.1 | 91.3 |
|  | **25** | 78.5 | 75.7 | 94.1 | 92.1 |
|  | **30** | 81.3 | 79.3 | 97.0 | 95.6 |
|  | **35** | 84.0 | 79.4 | 97.0 | 95.7 |
|  | **40** | 84.1 | 82.9 | 97.1 | 100 |
|  | **45** | 86.8 | 83.0 | 100 | 100 |
|  | **50** | 86.8 | 86.4 | 100 | 100 |
|  | **55** | 89.6 | 86.5 | 100 | 100 |
|  | **60** | 89.6 | 86.5 | 100 | 100 |
|  | **65** | 89.7 | 90.1 | 100 | 100 |
|  | **70** | 92.3 | 90.1 | 100 | 100 |
|  | **75** | 92.3 | 90.1 | 100 | 100 |
|  | **80** | 95.1 | 93.7 | 100 | 100 |
|  | **85** | 95.1 | 93.7 | 100 | 100 |
|  | **90** | 95.1 | 93.7 | 100 | 100 |
|  | **95** | 100 | 100 | 100 | 100 |

LFVFS: long-form visual functioning scale; SFVFS: short-form visual functioning scale; LFSES: long-form socio-emotional scale; SFSES: short-form socio-emotional scale.

**Table S4d: NEI VFQ-25 composite score and subscale scores in women without self-reported eye diseases aged 65-74 years of the German population-based Gutenberg Health Study (GHS), 2007-2012**

|  | | **LFVFS** | **SFVFS** | **LFSES** | **SFSES** |
| --- | --- | --- | --- | --- | --- |
| **N** | | 1266 | 1266 | 1266 | 1266 |
| **Mean** | | 83.7 | 81.5 | 94.7 | 93.8 |
| **SD** | | 12.1 | 13.0 | 9.4 | 10.6 |
| **Minimum** | | 3.7 | 4.4 | 28.6 | 24.6 |
| **Maximum** | | 100 | 100 | 100 | 100 |
| **Percentiles** | **5** | 61.8 | 57.7 | 74.7 | 70.5 |
|  | **10** | 67.7 | 65.0 | 83.3 | 79.6 |
|  | **15** | 70.6 | 68.5 | 88.2 | 85.7 |
|  | **20** | 75.7 | 72.1 | 91.2 | 91.1 |
|  | **25** | 76.2 | 75.7 | 94.1 | 91.3 |
|  | **30** | 78.7 | 75.8 | 94.2 | 95.6 |
|  | **35** | 81.3 | 79.3 | 97.0 | 95.7 |
|  | **40** | 84.0 | 82.1 | 97.0 | 95.7 |
|  | **45** | 84.6 | 83.0 | 97.1 | 96.4 |
|  | **50** | 86.8 | 86.4 | 100 | 100 |
|  | **55** | 89.1 | 86.5 | 100 | 100 |
|  | **60** | 89.6 | 86.5 | 100 | 100 |
|  | **65** | 89.6 | 90.1 | 100 | 100 |
|  | **70** | 92.3 | 90.1 | 100 | 100 |
|  | **75** | 92.3 | 90.1 | 100 | 100 |
|  | **80** | 92.3 | 92.8 | 100 | 100 |
|  | **85** | 95.1 | 93.7 | 100 | 100 |
|  | **90** | 95.1 | 93.7 | 100 | 100 |
|  | **95** | 97.2 | 96.4 | 100 | 100 |

LFVFS: long-form visual functioning scale; SFVFS: short-form visual functioning scale; LFSES: long-form socio-emotional scale; SFSES: short-form socio-emotional scale.
